# Indoor Air Radon Testing Rate and Its Relationships with Various Socioeconomic and Public Health Factors in Georgia, USA

**DOI:** 10.3390/ijerph23040450

**Published:** 2026-04-01

**Authors:** Uttam Saha, Kushajveer Singh, Derek Cooper, Pamela Turner, Rebecca Cantrell

**Affiliations:** 1 Agricultural and Environmental Services Laboratories, College of Agricultural and Environmental Sciences, The University of Georgia, 2300-2400 College Station Road, Athens, GA 30602, USA; kushajveer.singh@uga.edu; 2 Green Labs Program, University of Colorado Anschutz Medical Campus, 1945 North Wheeling Street, Aurora, CO 80045, USA; derek.cooper@cuanschutz.edu; 3 Department of Financial Planning, Housing and Consumer Economics, College of Family and Consumer Sciences, The University of Georgia, 224 Hoke Smith Annex, Athens, GA 30602, USA; prturner@uga.edu (P.T.); rebecca.cantrell@uga.edu (R.C.)

**Keywords:** indoor air, lung cancer, population density and diversity, public health, radon

## Abstract

**Highlights:**

**Public health relevance—How does this work relate to a public health issue?**

- Radon is a radioactive gas that is the leading cause of lung cancer among non-smokers and the second leading cause of lung cancer overall, claiming more than 21,000 lives annually in the USA; yet, the radon testing rate is generally low despite its significant public health consequences.
- Evaluating the association of radon testing rates with a broad range of demographic, socioeconomic, housing, literacy, and health indicators addresses a critical knowledge gap at the intersection of indoor air quality, environmental health, and social determinants of radon-related health consequences.

**Public health significance—Why is this work of significance to public health?**

- Identifies the important socioeconomic and public health factors that are primary determinants of indoor air radon-testing activities.
- Provides tools for identifying communities with systematically lower radon testing rates to support targeted, socio-culturally relevant public health outreach and indoor air quality interventions.

**Public health implications—What are the key implications or messages for practitioners, policy makers and/or researchers in public health?**

- Incorporating social determinants into environmental and public health surveillance and policy design is extremely important.
- Socio-culturally relevant outreach activities designed based on 49 socioeconomic and public health variables would be effective to increase testing rates and minimize the public health consequences of environmental radon.

**Abstract:**

Radon (^222^Rn_86_), the second leading cause of lung cancer, is common in indoor air. However, radon testing is generally low throughout the US. In this study, we utilized 134,496 short-term indoor air radon test results from Georgia, USA. We investigated the association of the radon testing rate with a total of 104 different independent variables belonging to seven categories: (1) Demographic and Neighborhood Characteristics; (2) Housing Characteristics; (3) Literacy and Numeracy; (4) Employment and Economy; (5) Selected Social Factors; (6) Access to Computer/Internet; and (7) Status of Healthcare, Health, Well-being, and Lifestyle. We used Bivariate Correlation, Multivariate Ordinary Least Squares (OLS) Regression, and Factor Analysis, followed by factor score-based OLS regression. Significant negative associations of the testing rates were observed with population diversity, residential segregation, urban population density, younger population, housing age, household size, low literacy, unemployment, childcare cost burden, poverty, obesity, and the frequency of mentally and physically unhealthy days. In contrast, testing rates were positively associated with older population, home value, owner-occupied homes, higher literacy, higher institutional education, income, prevalence of social association, and life expectancy. The findings provide valuable insights for identifying the communities where socio-culturally relevant outreach activities would increase testing rates and minimize the public health consequences of environmental radon.

## 1. Introduction

Radon (^222^Rn_86_) is a naturally occurring radioactive gas formed by the decay of uranium and radium in soil and bedrock. It is colorless, tasteless, and odorless, entering buildings through foundations and accumulating in indoor air. Radon exposure is responsible for approximately 21,000 deaths annually in the US, making it the second leading cause of lung cancer and the leading cause among nonsmokers [1,2].

Geological variation in radon potential results in significant geographic disparities in potential radon exposure across the United States. However, assessment of actual exposure risk predominantly relies on the radon testing rates. Georgia exhibits particularly diverse geologic features, with northern counties characterized by granite formations associated with potentially elevated radon emissions [3]. Many of these counties are classified as Zone 1 or Zone 2 by the EPA, indicating high or moderate radon hazard potential [4]. Geological radon hazard potential zones sometimes show a positive association with residential radon testing rates because people in the high-risk potential areas are generally more aware of the radon-related health effects due to more frequent radon-related outreach activities by state programs. It is also true that areas with low testing rates often mask high radon concentrations, leaving residents vulnerable, while proactive, higher testing rates are essential for uncovering, monitoring, and mitigating high indoor radon levels.

However, radon levels in any given home cannot be predicted based solely on geography or building characteristics. Testing represents the critical first step in identifying indoor radon exposure and initiating mitigation (if needed) to substantially reduce radon exposure [5]. Yet, national data indicate that only a minority of households pursue radon testing, and testing rates show a remarkable geographical disparity. Disparities in testing uptake may perpetuate inequities in exposure awareness and health outcomes among communities.

While substantial research has focused on geological predictors of radon potential, building characteristics influencing radon entry, and the technical performance of mitigation systems, comparatively less attention has been given to the social and structural factors that influence radon-testing behavior. Understanding the social determinants of exposure detection is increasingly recognized as essential for effective risk management. Indoor air quality is shaped not only by pollutant sources and building design but also by human behavior, access to resources, and community-level conditions. Identifying population-level patterns in radon testing can inform more equitable, targeted, and culturally relevant outreach strategies, thereby improving indoor environmental quality and reducing preventable health risks. Emerging evidence suggests that radon testing rates may vary systematically according to demographic composition, housing tenure, education, income, and access to information or health resources [6,7]. Communities characterized by lower socioeconomic status, higher residential mobility, or limited health literacy may face barriers to radon awareness, testing, and mitigation, even when the underlying radon potential is high. Another study suggests that income, educational attainment, homeownership, and geographic isolation influence radon-testing behavior, which is linked to environmental health literacy, financial resources, and access to mitigation services [8]. However, most studies in this regard examined a narrow set of predictors or relied on small geographic areas, limiting their ability to capture the complex, multidimensional drivers of testing behavior.

Georgia has a remarkable demographic diversity and pronounced variation in socioeconomic and public health scenarios. Some of the state’s highest-radon counties are also among its poorest and most rural, raising concerns about environmental health inequity. Also, a large, long-term dataset of indoor radon test results for Georgia is available through the State Indoor Radon Program contributed by the testing laboratories. All of these provide a unique landscape to examine the relationships between the important factors and people’s radon-testing behavior. By linking radon testing rates with a broad range of demographic, socioeconomic, housing, literacy, and health indicators, this study addresses a critical knowledge gap at the intersection of indoor air quality, environmental health, and social determinants of health.

The primary objective of this study was to examine county-level associations between indoor radon testing rates and a comprehensive set of demographic, socioeconomic, housing, literacy, and health-related factors in the state of Georgia, United States. Specifically, this study aimed to: 

(1) Quantify indoor radon testing rates per 1000 occupied housing units using short-term radon test data collected between 1990 and 2022.

(2) Evaluate bivariate associations between radon testing rates and 104 independent variables across seven domains: demographic and neighborhood characteristics; housing characteristics; literacy and numeracy; employment and economy; selected social factors; access to computer and internet; and status of healthcare, health, well-being, and lifestyle.

(3) Identify underlying patterns among predictors using factor analysis and assess the dependence of radon testing rates on the predictors using appropriate regression models.

(4) Identify communities with systematically lower radon testing rates to support targeted, socio-culturally relevant public health outreach and indoor air quality interventions. 

(5) Identify patterns relevant to public health policy and conduct socio-culturally relevant outreach activities that would increase testing rates and minimize the public health consequences of environmental radon.

We hypothesize that (1) counties with higher socioeconomic status (income, education, homeownership, etc.) will show higher radon testing rates; and (2) counties with higher smoking prevalence and other awkward public health indicators will not have correspondingly higher radon testing rates (creating pockets of elevated combined lung cancer risk). These hypotheses are grounded in a prior limited-scale study in DeKalb County, Georgia, which found geographically uneven testing rates, and the low testing rates across the county were significantly driven by residential segregation, which expanded over 25 years; however, the associations of radon testing rates with the other social indicators, such as income or education, were weaker [9].

## 2. Materials and Methods

### 2.1. Indoor Air Radon Test Data

Each year, the University of Georgia (UGA) radon program requests radon test data on short- and long-term radon test kits from major radon laboratories. These include Air Chek, Pro Lab, First Alert, AccuStar, and Dr. Home Air and Alpha Energy Laboratories. Residents voluntarily chose to test their dwellings with either envelope- or canister-type test kits, which are available from the state radon program, home supply or big box stores, or online. Both types of test kits contain activated charcoal, which absorbs radon. Envelope-type kits are hung on interior walls for 3–7 days, whereas canister-type test kits are placed on countertops or other surfaces for 2–4 days according to the ANSI/AARST-MAH standard [10]. After the duration of the test, the kits are shipped to manufacturers’ designated laboratories for radon analysis. The required decay correction is made to determine the final radon concentration. The average differences between the two types of short-term test kits are insignificant and have been reported as 27 Bq/m^3^ (0.73 pCi/L) by Dai et al. [11].

**Figure 1:**
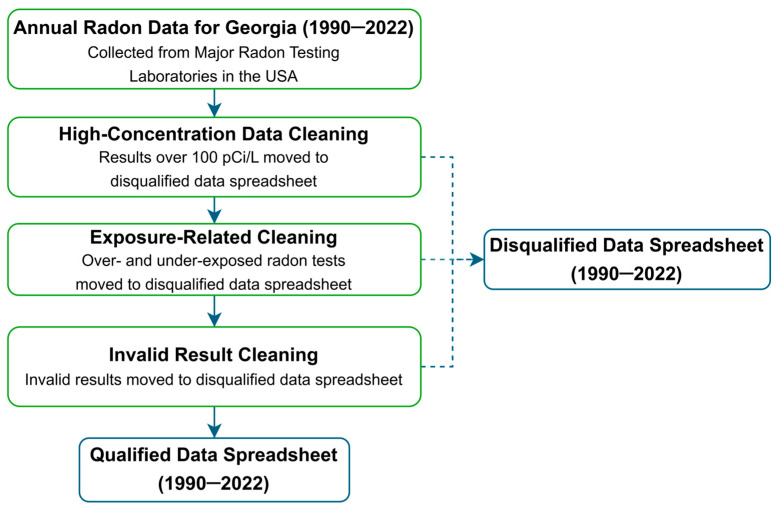
Collection, compilation, and handling of indoor air radon test results for Georgia, 1990–2022.

Figure 1 depicts the radon testing data collection, compilation, and handling procedure followed in this study. Testing laboratories report test results to the state program as requested each year. Included in the reports is information containing zip code, county, city, and test results, which are measured in picocuries per liter of air (pCi/L). This data is anonymized and does not contain identifying information. In this study, a large database of indoor air radon test results for the state of Georgia was built. The results were from voluntary tests conducted during 1990–2022. After excluding ambiguous/disqualified results, the database had a total of 134,496 test results. The data were segregated by 159 counties of the state of Georgia. A detailed description of these radon tests’ data collection, compilation, and exclusion of outliers is available elsewhere [3].

**Figure 2:**
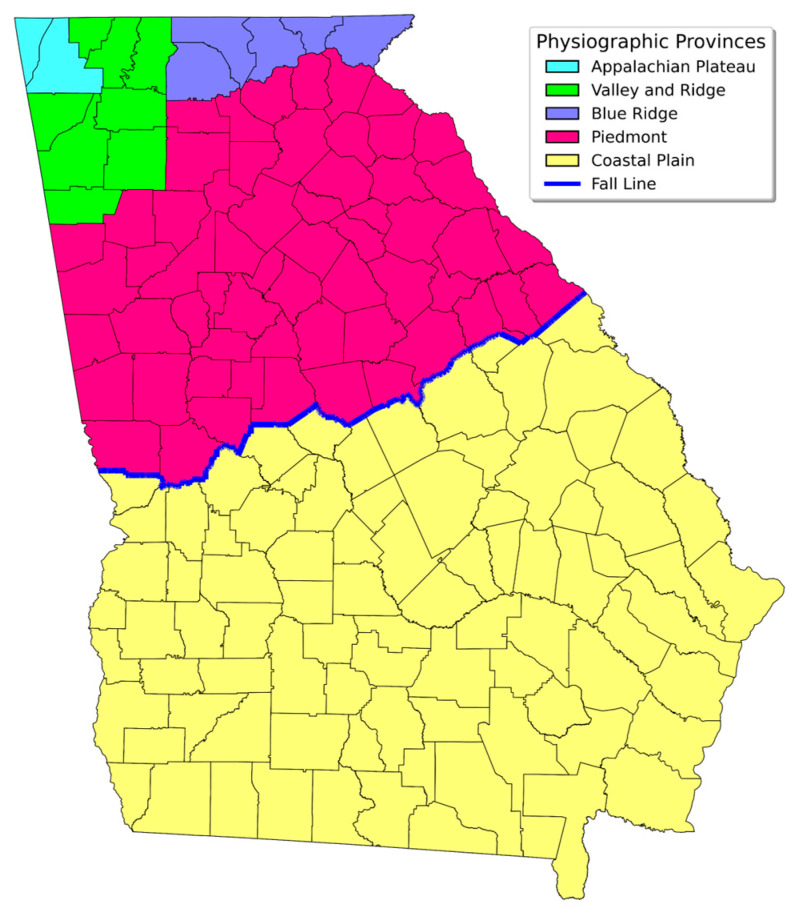
Georgia’s physiographic provinces and the Fall-Line.

The state of Georgia is divided into 5 physiographic provinces: Appalachian Plateau (APP), Valley and Ridge (VR), Blue Ridge (BR), Piedmont (P), and Coastal Plain (CP). Between the P and CP regions, there is a 20-mile-wide “Fall-Line” across the middle of the state (Figure 2). This is a geological boundary, a gently sloping region that rapidly loses elevation from the north to the south, thereby creating a series of waterfalls. During the Mesozoic Era (25.1–65.5 million years ago), the Fall-Line was the shoreline of the Atlantic Ocean. Today, it separates the upper CP to the south from P to the north. The area above the Fall-Line sits on various crystalline rocks, whereas the area below the Fall-Line has up to 7000 feet of unconsolidated sediments of marine origin. In our previous study, it was revealed that the chances of obtaining equal to or greater than 4.0 pCi/L (the current action limit of USEPA) radon test results were 8–10 times higher in the area above the Fall-Line than in the area below the Fall-Line, which was primarily due to the contrasting underground geological features of the two regions [3]. From a radon exposure and public health protection standpoint, it is very important to increase the indoor air radon testing rates, especially in the area above the Fall-Line with higher radon potential. Identifying the socioeconomic and public health variables that could contribute to radon testing decisions by the homeowners in this area would be instrumental to increasing the testing rates. Therefore, we limited this study to a total of 106,264 test results (testing period: 1990–2022) from the 73 counties above the Fall-Line with higher indoor air radon potential.

### 2.2. Socioeconomic and Public Health Data Collection

This study investigated the association of radon testing rates with a total of 104 different independent variables belonging to 7 categories: (1) Demographic and Neighborhood Characteristics; (2) Housing Characteristics; (3) Literacy and Numeracy; (4) Employment and Economy; (5) Selected Social Factors; (6) Access to Computer/Internet; and (7) Status of Healthcare, Health, Well-being, and Lifestyle. County-level data for these variables for the state of Georgia were collected from the following reliable sources:

1. Demographic, housing and occupancy characteristics, home value, income and poverty data were downloaded from the US Census Bureau in tabular forms (available online at: https://data.census.gov/table, accessed on 15 September 2025). These data were available for the year 2000, and for each year from 2010 to 2022. These 14-year data showed some temporal change over the years, especially in the counties in the metro Atlanta area.
2. Literacy, numeracy, different tiers of institutional education, employment status, types of occupation, and poverty data were downloaded from the website of the Program for the International Assessment of Adult Competencies (PIAAC) under the National Center for Education Statistics under the US government (https://nces.ed.gov/surveys/piaac/state-county-estimates.asp, accessed on 22 September 2025). Most of these data were available for the years 2013 to 2017. This dataset did not show any remarkable temporal change over the 5-year period.
3. Data for various public health variables were downloaded from the County Health Rankings and Roadmaps (CHR&R) website, University of Wisconsin Population Health Institute (https://www.countyhealthrankings.org/, accessed on 29 September 2025). These data were available for each year from 2011 to 2022. There were hardly any remarkable changes in this dataset over the years.

The median numeric data for any given socioeconomic and public health variable for each county were used as the independent variables, which were hypothesized to have potential effects on the radon testing rates (the dependent variable). Median instead of mean values were used because median provides a robust measure of central tendency that is known for resistance to outliers and skewness.

The radon test data used in this study spanned from 1990 to 2022, but the socioeconomic and public health variables were not exactly from the same period as described above. Thus, the use of the temporal median values of these socioeconomic and public health variables to predict the radon testing patterns could be viewed as a potential limitation of this study. It is important to mention here that the variables listed under #2 and #3 above had hardly any remarkable temporal changes; however, the ones listed under #1 did have some temporal changes, especially for the counties in the metro Atlanta area. Nevertheless, it should be considered as the best effort to accomplish the study objectives based on the available data within the 1990 to 2022 time range.

### 2.3. Data Analysis

The radon-testing activities in the 73 counties above the “Fall-Line” were evaluated using the testing rates per 1000 occupied housing units rather than the actual number of tests because both the number of tests and the number of occupied housing units varied widely for the counties. To address spatial (county) variance, this study used a Spatial Empirical Bayes (SEB) method [12,13,14] of smoothing radon testing rates. This method was chosen because the small number of tests in some counties may make the crude testing rates fluctuate, which potentially may erroneously suggest low testing outliers [13]. The SEB method implemented relied on the spatial weights to estimate testing rates at each county by including testing observations in the neighboring counties within the study area (i.e., the area above the Fall-Line). For each county, this study used all immediate neighboring counties in its spatial weight calculation. This smoothing helped reduce variance in the radon testing rates in any given small area within the study area while preserving the local spatial variations.

The numeric ranges of the data for different socioeconomic and public health variables (104 in total) were widely different, and they were on different units. Therefore, each of these datasets was standardized by dividing the absolute difference between the individual datapoint and the mean value by the standard deviation, essentially producing the Z-scores (or standardized scores). This makes different datasets comparable by centering them at a mean of 0 and a standard deviation of 1 (z-scaling). This essentially prevents variables with larger numeric values from exerting a dominant effect on the outcomes of statistical analysis (biased outcomes).

Using bivariate correlation and multivariate regression models, this study evaluated how radon testing rates were correlated with 104 different socioeconomic and public health variables. The initial assessment started from a bivariate correlation analysis between the radon testing rates (dependent variable) and each of the 104 socioeconomic and public health variables (the independent variables). The 55 out of 104 independent variables that had significant (at *p* ≤ 0.05) bivariate correlation coefficients with the dependent variable were taken for further analysis. First, a bivariate correlation matrix among these 55 independent variables with 1485 correlation coefficients was calculated to evaluate the presence of multicollinearity among them. The subsequent multivariate ordinary least squares regression analysis was carried out using the radon testing rate as the dependent variable and all 55 independent variables. The multiple regression analysis used in the study is one of the methods to describe the relationship between one dependent variable (radon testing rate) and multiple independent variables (55 socioeconomic and public health variables). Equation (1) below is the general expression of the multiple regression model used.

Y = β_0_ + β_1_X_1_ + β_2_X_2_ + ………………….. + β_55_X_55_ + ε (1)

In Equation (1),

Y is the dependent variable;

X_1_, X_2_, …….. X_55_ are the 55 independent variables;

β_1_, β_1_, …….. β_55_ are regression coefficients;

ε is the error term.

Both radon testing rate data and many socioeconomic and public health variables were not normally distributed. Therefore, logarithmic transformation was applied to X and Y as applicable (or required) for meeting the underlying assumption of multivariate ordinary least squares regression analysis.

**Figure 3:**
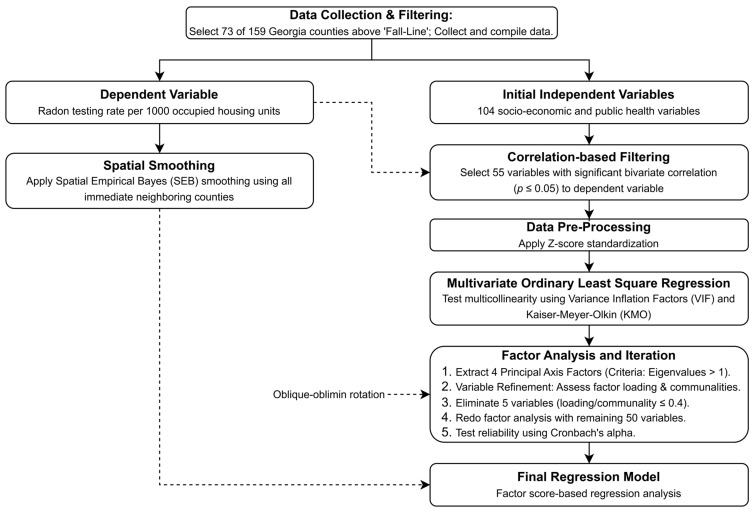
Flowchart showing data analysis protocol.

The Variance Inflation Factors (VIF) and Kaiser–Meyer–Olkin (KMO) statistic were calculated and evaluated to determine if the presence of multicollinearity among the 55 independent variables affected the regression analysis. The multicollinearity issue was finally resolved through factor analysis followed by regression that used factor scores as the independent variables. Factor analysis evaluated the underlying structure of the 55 independent variables and then determined the best fit between these related variables and the latent uncorrelated factors. It initially used principal axis factoring (PAF) for extracting the factors from the dataset, which focused on shared variance and is robust to non-normality. The PAF returns as many components (factors) as variables while capturing much of the variance from the data. The method then retained the principal axis factors that had an eigenvalue greater than one in the Scree plot. For computing loadings, we used oblique-simplimax rotation rather than any orthogonal rotations because we found that a vast majority of the 55 independent variables were significantly correlated with each other. This technique maximized the loading of a variable on one factor and minimized its loadings on all others. In the first round of factor analysis, all 55 independent variables were included, and their factor loadings (correlation with a factor) and communalities (variance explained) were examined. In this step, 5 variables with factor loading and/or communality ≤ 0.4 were identified and excluded to differentiate significant, stable variables from those that contribute mostly noise, thereby ensuring that the remaining 50 factors after exclusion of the 5 are reliable, interpretable, and adequately represent the underlying data structure. Thus, the final round of factor analysis was carried out with these remaining 50 variables. Once a suitable number of factors was determined and loading scores for each factor were computed, the final regression analysis of the dependent variable against the loading scores of the factors (independent variable) was carried out. The reliability of the factors and factor scores-based regression analysis was confirmed based on the standardized Cronbach’s alpha statistic for each factor. The flowchart of the statistical analyses used in this study, as described above, is presented in Figure 3.

## 3. Results

### 3.1. Number and Rates of Radon Tests

**Figure 4:**
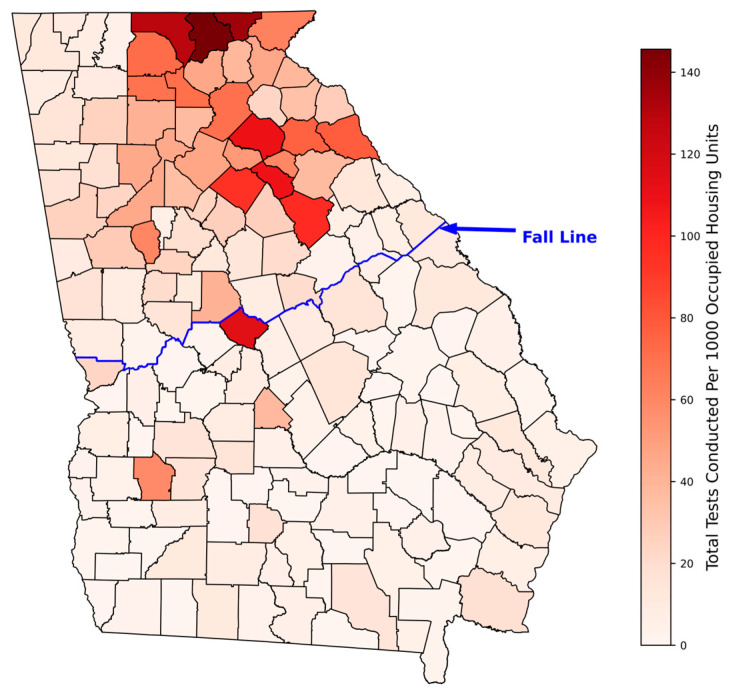
Total number of radon tests conducted in all 159 Georgia counties during 1990–2022.

**Figure 5:**
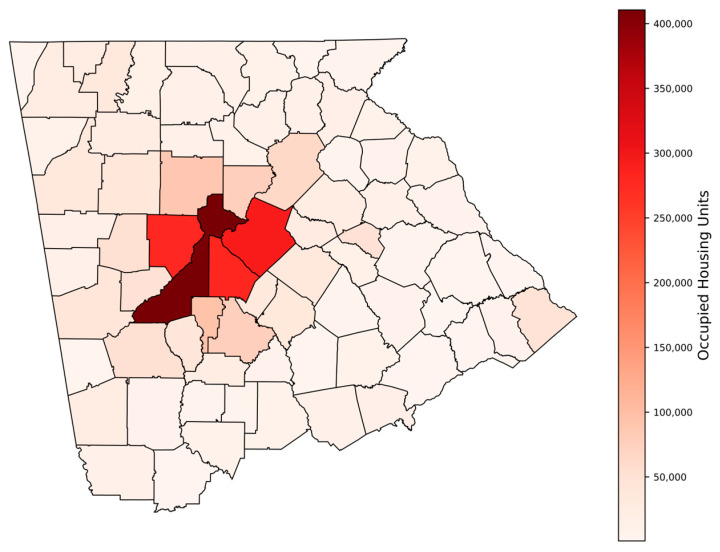
Number of occupied housing units in the 73 Georgia counties above the Fall-Line (the study area).

**Figure 6:**
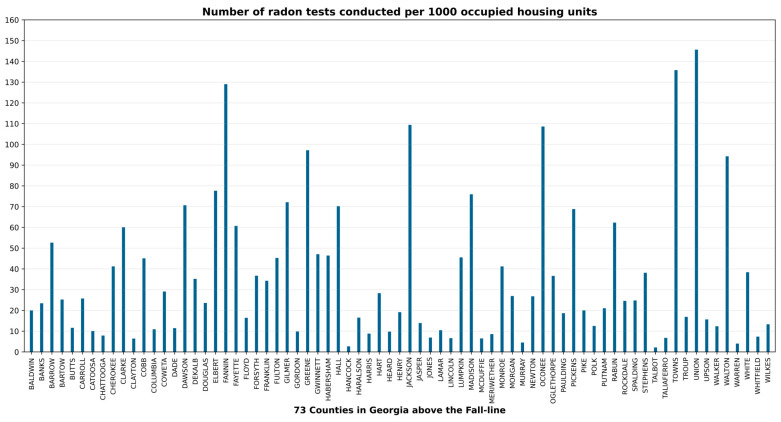
Number of radon tests conducted per 1000 occupied housing units (testing rates) in the 73 Georgia counties above the Fall-Line (the study area).

Figure 4 shows the total tests conducted in all 159 counties of Georgia and the Fall-Line dissecting the state. The area above the Fall-Line, which is the study area of this paper, sits on various crystalline rocks, whereas the area below the Fall-Line has up to 7000 feet of unconsolidated sediments of marine origin. Statewide, the total tests conducted in the 159 counties varied from 0 (none) to 18,592, with a mean of 760 ± 2318. Likewise, in the study area, the total number of tests in the 73 counties varied widely from 1 to 15,418, with a mean of 1134 ± 2589. The number of occupied housing units in the 73 counties ranged from as low as 593 to as high as 410,576, with a mean of 37,722 ± 71,955 (Figure 5). The testing rate per 1000 housing units in the 73 counties of the study area also varied widely from 2 to 146, with a mean of 36 ± 34 (Figure 6).

### 3.2. Bivariate Correlation of the 104 Independent Variables with the Radon Testing Rates

We found that 55 out of 104 independent socioeconomic and public health variables had significant correlation coefficients with the dependent variable, radon testing rates (Table 1). Out of these 55, 35 correlation coefficients (r > 0.281) were significant at *p* ≤ 0.01 and the remaining 20 (r = 0.230 to 0.281) at 0.01 ≤ *p* ≤ 0.05. The description of these 55 independent variables and their actual correlation coefficients (r), along with their statistical significance (*p*-value), are presented in Table 2. These 55 independent variables were taken for further statistical analysis and the other 49 were excluded.

### 3.3. Correlation Matrix Among the 55 Independent Variables

As evident in Table 3 and Table 4, there was substantial multicollinearity among the 55 independent variables that had significant bivariate correlations with the dependent variable (number of radon tests per 1000 occupied housing units). Out of 1485 bivariate combinations, 1015 had correlation coefficients, r > 0.281 with *p* ≤ 0.01, and 76 had r = 0.230–0.281 with 0.01 ≥ *p* ≤ 0.05, demonstrating the fact that these variables are frequently intercorrelated, which could affect the usual multiple linear regression analysis (Table 4).

### 3.4. Multivariate Least Squares Regression Analysis of Radon Testing Rates (Independent) Versus 55 Independent Variables

The multivariate ordinary linear least squares (OLS) regression analysis results are presented in Table 5. The Jarque–Bera (JB) statistic in the multivariate regression analysis of radon testing rates (dependent variable) *versus* 55 independent variables was low (0.93), with a high *p*-value (0.63), meaning the errors were likely to be normally distributed. This ensured that OLS estimators were themselves normally distributed (multivariate normal), allowing for valid statistical inference like hypothesis testing, though a fairly large sample size in this study could bypass the strict normality requirement due to the Central Limit Theorem, making OLS a Best Linear Unbiased Estimator (BLUE) regardless. While the R-squared value (0.945) indicated that as much as 94.5% of the total variance was explained by all 55 predictors, the adjusted R-squared value (0.444) was greatly reduced. This suggests that there was a continued increase in R-squared with added predictors, whereas adjusted R-squared increased only if the added predictors significantly improved the model, otherwise it decreased. The observed large difference between R-squared and adjusted R-squared suggests that there was probably a good number of less useful predictors in the regression model. As such, the F-statistic (1.885) was not significant, with a *p*-value of 0.217. Furthermore, the Omnibus statistic (1.657) was insignificant, with a *p*-value of 0.437, suggesting that the 55 predictors collectively did not explain a significant amount of variance in the outcome, essentially revealing that the model was not better than baseline (no predictors), i.e., an overall unsatisfactory model fit. The Durbin–Watson (DW) statistic for checking for autocorrelation (serial correlation) in the model’s residuals was 2.11. As a rule of thumb, a DW statistic between 1.5 and 2.5 generally suggests that autocorrelation is not a major concern. The prediction coefficients for all 55 independent variables were statistically not significant, with *p* > |t| ranging from 0.09 to 0.99.

Variance Inflation Factor (VIF) was invariably much higher than 10 and the smallest eigenvalue was close to zero (1.26 × 10^−29^). Furthermore, the Kaiser–Meyer–Olkin (KMO) statistic was high (0.86). All of this suggests that there was a substantial multicollinearity among the 55 predictor variables, usually a problem for OLS, which led the OLS regression results presented in Table 5 to be less reliable. However, these observations are valuable because they suggest that the patterns of correlations among the predictor variables are relatively compact, and this correlation is highly appropriate for reduction through structure-detection techniques like factor analysis [15]. This should yield distinct and reliable factors and reduce the dimensionality before running a multivariate ordinary least squares (OLS) regression and thereby reducing potential issues with multicollinearity in the regression model. This means that the large number of correlated predictor variables can be successfully reduced into a few more manageable factors using a suitable statistical protocol. The scores of these factors can then be used in multivariate OLS regression, which would reliably model the dependent variable using a single set of predictors and yield a robust, reliable, and interpretable multivariate OLS regression model.

### 3.5. Factor Analysis and Factor Score-Based Regression Analysis

**Figure 7:**
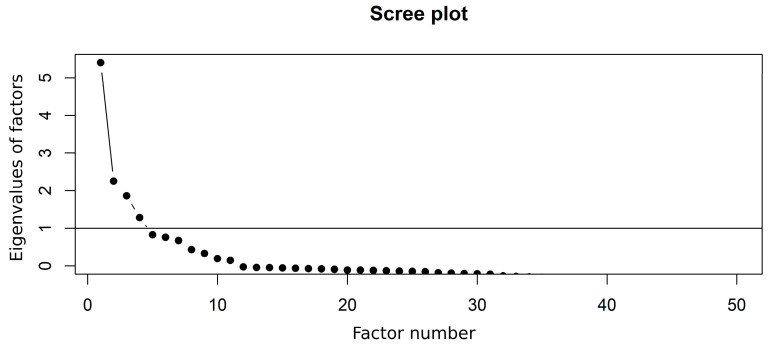
Scree plot obtained in factor analysis showing 4 factors with eigenvalues greater than 1.0. Black dots (•) are the eigenvalues of the factors.

The results of factor analysis revealed that the eigenvalues of the four factors were greater than 1 (Figure 7), suggesting that the first four factors were suitable for use as independent variables in the regression analysis. The selected four factors explain the most variance (0.988) for “More_HS (proportion of population with education beyond high school)” and the least (0.425) for “Primary Care Physician (PCP) rate (#PCP per 100,000 population)” (Table 6).

The first, second, third, and fourth factors explained 57.89, 11.10, 7.94, and 4.41% of the total variance in all variables, respectively, adding to a total of 81.35% (Table 6). Separately, the first factor explained 71.17% [(28.847 ÷ 40.674) × 100] of the variance, while the second, third, and fourth factors explained 13.65, 9.76, and 5.42% of the variance, respectively. It was possible to calculate Cronbach’s alpha (standardized) values for the first, second, and third factors only, but not for the fourth factor, because it had only one variable (% of owner-occupied housing units). Cronbach’s alpha values for the first three factors were high, ranging from 0.818 to 0.988 (Table 6), confirming that the grouping of the independent variables into factors by factor analysis implemented in this study was reliable and their scores were suitable for regression analysis. The factor loading scores given in Table 6 show the relationship between the independent variables investigated and the factors. The bold values in the table show the highest correlation between the variables investigated and factors. The absolute values of the factor loadings of the 40 independent variables included in the first factor ranged from 0.473 to 0.990; the same of the five variables in the second factor, four variables in the third factor, and one variable in the fourth factor were from 0.649 to 0.857, 0.568 to 0.783, and 0.520, respectively.

The regression analysis results (Table 7) of the radon testing rates (the dependent variable) versus the factor scores (the independent variables) of the four factors show that the regression coefficients of Factor-1, Factor-2 and Factor-3 were statistically significant (*p* < 0.05), whereas that of Factor-4 was not significant (*p* = 0.275). The F-statistic was highly significant with a very low *p*-value of 8.94 × 10^−6^. The VIFs for the four factors were invariably lower than 10, ranging from 1.77 to 7.68. Thus, the observed multicollinearity issue among the socioeconomic and public health variables was properly resolved by factor analysis followed by factor score-based regression. The 40 dependent variables in the first factor, five in the second factor, and four in the third factor appeared as the significant determinants of the radon-testing activities.

## 4. Discussion

This study provides a comprehensive, multi-scale assessment of indoor radon testing patterns in the 73 high-risk counties above the Fall-Line and demonstrates that testing rates are strongly structured by socioeconomic and public health contexts. The findings advance the environmental health literature by showing that radon testing—a prerequisite for exposure reduction—is not simply a function of geologic risk but is deeply embedded within broader social determinants of health. Radon testing rates were higher in areas characterized by greater household income, higher literacy–numeracy–educational attainment, higher rates of homeownership, newer homes, higher home value, access to computer and internet, social association, availability of Primary Care Physicians, and access to healthy foods. In contrast, testing activities were lower in the counties with higher population diversity and residential segregation, urban population density, unemployment, childcare cost burden, poverty, frequency of Food Stamps/SNAP recipients, and single-parent households. These associations likely reflect differences in risk awareness, financial capacity to purchase test kits and mitigation services, and housing stability, which incentivize long-term investment in indoor environmental quality. Importantly, the persistence of these associations in multivariate models suggests that socioeconomic advantage independently facilitates engagement with radon-testing behaviors, reinforcing concerns that voluntary testing frameworks may systematically underserve lower-income, less-educated, and disadvantageous communities.

A previous study by Dai [9] demonstrated that in DeKalb County, Georgia, United States, the areas with lower residential segregation index between white and Black populations had substantially higher socioeconomic advantages in terms of bachelor’s degrees, median housing value, and median income and had 2 to 8 times greater radon testing rates, which was substantiated by subsequent bivariate analysis, establishing the consistent influence of higher-education level, i.e., bachelor’s degrees, and socioeconomic advantages on radon screening rates. Therefore, the observed effect of segregation on radon screening decisions was partially mediated by educational and socioeconomic differences. Multiple studies have reported the adverse effects of residential segregation and associated socioeconomic disadvantages on public health [16,17,18], yet little is known about their consequences on environmental threats that could lead to adverse health effects. Darden et al. [19] and White and Borrell [20] reported that communities with disadvantageous socioeconomic barriers, such as poor education and low income, could affect public awareness of radon, leading to lower radon-testing activities. People’s socioeconomic constraints limit their potential decision-making and thus may affect health-promotion behaviors [21,22]. Socioeconomic disadvantages may directly influence radon knowledge and the health risks homeowners perceive, which in turn lowers testing participation. Even though the area of this study has higher radon potential in Georgia, we found the recurring problem of generally low and spatially heterogeneous testing activities as reported from several studies in other states or countries [23,24,25]. Widespread testing is less likely with poorer radon knowledge and risk perception because radon testing is voluntary in the United States. Despite outreach education activities through State Indoor Radon Programs for the past many years and abundant online resources on various aspects of radon, testing remains as low as 3% to 9% of homes nationwide [26,27,28]. While awareness about radon is often considered the principal determinant of taking a test, as many as half of the homeowners in the low testing areas may not conduct tests even with their awareness [29], warranting investigation into other factors affecting radon-testing behaviors. The findings of this study highlight the importance of target-oriented radon awareness and educational campaigns not just based on the geologic potential of radon but also incorporating socioeconomic and other variables affecting radon-testing activities. As observed in previous studies [9,30], we also found that communities with lower literacy, numeracy and institutional education and with economic disadvantages indicated by employment and poverty were negatively associated with testing rates. Thus, this study makes a key contribution in exposing county-level correlations of various demographic, housing, educational, and socioeconomic variables with the testing rates for indoor radon.

Public health indicators also emerged as significant determinants of radon-testing activity. Areas with higher obesity, percentage of physically inactive population, percentage of adults reporting fair or poor health, Primary Care Physician (PCP) ratio (number of people served by one PCP), percentage of population with excessive drinking, average number of physically or mentally unhealthy days per month, smoking prevalence, and age-adjusted lung and bronchus cancer incidence rate cases per 100,000 people exhibited lower testing rates. The observed inverse relationship of radon testing rate and age-adjusted lung and bronchus cancer incidence rate appears reasonable because people unknowingly living with high radon exposure are susceptible to a higher risk of developing lung and bronchus cancer. However, it is remarkable and unexpected that the health-wise more vulnerable communities with higher smoking prevalence and other precarious public health situations are less likely to consider radon testing, thereby exposing them to more severe health consequences of indoor air radon. Given the well-established synergistic effect between radon exposure and smoking on lung cancer risk, the observed inverse association between smoking prevalence and testing rates is particularly concerning. This pattern implies that populations facing compounded lung cancer risk may simultaneously experience a lower likelihood of radon risk detection, exacerbating existing health inequities.

A key contribution of this study is the identification of geographic mismatches between radon potential and testing activity. Several counties with moderate to elevated radon potential demonstrated persistently low testing rates due to the socioeconomic and public health factors discussed above, indicating potential under-recognition of environmental risk. From a public health perspective, these areas represent high-priority targets for intervention, as undetected exposure may lead to disease burden, which could be preventable by radon testing and mitigation. The results underscore the limitations of relying on geologic risk maps alone and highlight the importance of integrating social and public health vulnerability metrics into radon surveillance and outreach strategies. The observed spatial disparities in radon testing suggest that existing public outreach interventions based on geologic potential have not substantially altered the underlying social gradient in radon testing. This finding aligns with broader environmental health research, indicating that information-based voluntary testing often yields uneven benefits unless explicitly designed to address structural barriers related to socioeconomic and public health scenarios.

Taken together, the findings position radon testing as both an environmental exposure issue and an indicator of socioeconomic and public health equity. The results support calls for more proactive, equity-oriented radon control strategies, including subsidized or free testing in high-risk and underserved areas, the integration of radon education into primary care and smoking cessation programs, strengthening real estate disclosure requirements, and partnerships with local housing and community organizations. By reframing radon testing as a public health service rather than an individual consumer choice, policymakers may better address the social, public health, and geographic inequities documented in this study. While this analysis focuses on the high-risk areas above the Fall-Line in Georgia, the mechanisms identified—linking socioeconomic conditions, public health situations, and environmental risk detection—are likely applicable to other states with heterogeneous radon potential and decentralized testing policies. As such, the findings contribute to a growing evidence base supporting the incorporation of social determinants and public health variables into environmental radon exposure prevention and lung cancer control strategies.

## 5. Conclusions

Indoor radon testing in Georgia is unevenly distributed and closely linked to a number of socioeconomic and public health variables. The results of the study showed that there was a significant multicollinearity among various socioeconomic and public health variables, which were used as the potential determinants of the radon testing rates in the radon-prone areas of Georgia, US. Thus, instead of directly using these variables, the use of the factor analysis scores obtained from the variables reduced the risk of inaccurate interpretations of the parameters in the model according to the least squares method. Furthermore, using comparison, the study showed the applicability of the regression analysis results by using the classical, least squares method-based multiple linear regression *versus* factor analysis scores-based regression analysis in the case of multicollinearity among independent variables. Out of 104 socioeconomic and public health variables evaluated, 49 appeared as the reliable determinants of the radon-testing activities in the study area. Socio-culturally relevant outreach activities designed based on these 49 variables would be effective to increase testing rates and minimize the public health consequences of environmental radon. Thus, this study highlights the importance of incorporating social determinants into environmental health surveillance and policy design.

### Limitations

As stated in Section 2.2, there was a temporal mismatch between the radon test data and various socioeconomic and public health variables. However, it should be considered as the best effort to accomplish the study objectives based on the available data within the 1990 to 2022 time range. This study used the temporal median values of various socioeconomic and public health variables as a pragmatic approach to create a stable, cross-sectional profile of the counties because median provides a robust measure of central tendency that is resistant to outliers and skewness.

This study relies on reported radon tests, which may underestimate true testing activity. Ecological analyses cannot infer individual behavior, and some variables may be subject to measurement error. Nevertheless, the integrated, multi-scale approach provides valuable insights into population-level patterns.

## Figures and Tables

**Table 1:** Summary of bivariate correlation between the dependent variable (number of radon tests conducted per 1000 occupied housing units) and 104 socioeconomic and public health independent variables.

Correlation Coefficient, r (Absolute Value)	Number of Bivariate Combinations	Significance of Correlation Coefficient, r
>0.281	35	*p* ≤ 0.01, 71 (73-2) df
0.230–0.281	20	0.01 < *p* ≤ 0.05, 71 (73-2) df
<0.230	49	*p* > 0.05, 71 (73-2) df
**Total** ** ^§^**	**104**	

**^§^ **Total number of bivariate combinations between the dependent variable and various socio-economic and public health independent variables.

**Table 2:** Bivariate correlation coefficients between the number of radon tests per 1000 occupied housing units (dependent variable) and various independent socioeconomic and public health variables (for the sake of brevity, only 55 out of 104 independent variables with significant correlation coefficients are shown).

Parameter # ^†^	Parameter Description	*Correlation Coefficient, r* ^§^	*p*-Value at 71 (73-2) df
	1.0 Demographic & Neighborhood Characteristics		
19	Population diversity index	**−0.3072**	0.0092
22	Residential segregation index: between Black & white populations	**−0.4087**	0.0003
23	Median age of the population	**0.3573**	0.0019
24	% of population below 18 years of age	**−0.3561**	0.0020
25	% of population 65 years and older	**0.4238**	0.0002
29	Urban population density (per sq. miles)	**−0.2537**	0.0303
	2.0 Housing Characteristics		
37	% of occupied housing units	**0.2328**	0.0475
39	% of owner-occupied housing units	**0.2645**	0.0237
40	Median home value ($)	**0.4996**	0.0001
43	% of housing units built 1970 or earlier (older homes)	**−0.4205**	0.0002
45	% of housing Units built 1990 or later (newer homes)	**0.3882**	0.0007
46	Average household size of owner-occupied units	**−0.2338**	0.0474
47	Average household size of renter-occupied units	**−0.2461**	0.0358
50	% of single-family homes detached	**0.2559**	0.0288
52	% of single-family homes, total: detached + attached	**0.2645**	0.0237
55	% of units in mobile homes	**−0.2360**	0.0444
	3.0 Population Literacy and Numeracy		
57	Lit_P1 (proportion of population with literacy level P1 and below)	**−0.4354**	0.0001
58	Lit_P2 (proportion of population with literacy level at P2)	**−0.2890**	0.0132
59	Lit_P3 (proportion of population with literacy level P3 and above)	**0.4105**	0.0003
60	Proportion of population with literacy level P2 + P3 combined	**0.4365**	0.0001
61	Lit_A (literacy average score indirect estimates)	**0.4085**	0.0003
62	Num_P1 (proportion of population with numeracy level P1 and below)	**−0.4316**	0.0001
64	Num_P3 (proportion of population with numeracy level P3 and above)	**0.3981**	0.0005
65	Proportion of population with numeracy level P2 + P3 Combined	**0.4335**	0.0001
66	Num_A (numeracy average score indirect estimates)	**0.4074**	0.0003
67	Less_HS (proportion of population with education less than high school)	**−0.3665**	0.0014
68	HS (proportion of population with high school education)	**−0.2904**	0.0127
69	More_HS (proportion of population with education beyond high school)	**0.3581**	0.0019
70	% of those with some college education	**0.2331**	0.0471
72	% of those with a bachelor’s degree in population 25 years and older	**0.2701**	0.0208
	4.0 Employment and Economy		
76	Unemployment (proportion of population aged 16–64 who are unemployed)	**−0.2378**	0.0428
78	Occupation: management (proportion of population aged 16 and over who are in the labor force in management, business, science, and arts occupations)	**0.3062**	0.0084
79	Median household income	**0.2546**	0.0297
81	Childcare cost burden (% of household income spent/required for childcare expenses)	**−0.3929**	0.0006
82	% of children in poverty	**−0.2541**	0.0301
84	Poverty_100 (proportion of population who are below 100 percent of the poverty level)	**−0.2625**	0.0249
85	Poverty_150 (proportion of population who are below 150 percent of the poverty level)	**−0.2898**	0.0129
87	SNAP (proportion of households that have received Food Stamps/SNAP in the past 12 months)	**−0.3884**	0.0007
	5.0 Social Factors		
92	Resilience: % of population with 1–2 risk factor	**0.2479**	0.0345
96	% of children in single-parent households	**−0.2722**	0.0198
97	Social association rate (# of social associations per 10,000 population)	**0.2369**	0.0436
	6.0 Internet Access		
94	% of households with a computer	**0.2311**	0.0491
95	% of households with broadband internet subscription/access	**0.3327**	0.0040
	7.0 Healthcare, Health, and Well-being		
100	Primary Care Physician (PCP) rate (#PCPs per 100,000 population)	**0.3274**	0.0047
101	Primary Care Physician (PCP) ratio (#people served by one PCP)	**−0.3822**	0.0008
103	Life expectancy	**0.3918**	0.0006
104	Food environment index (indicator of access to healthy foods: 0 is the worst, 10 is the best)	**0.3276**	0.0047
105	% of adults with obesity	**−0.4751**	0.0001
106	% of population physically inactive	**−0.4604**	0.0001
107	% of population with excessive drinking	**−0.3573**	0.0019
108	% of adults who reported currently smoking	**−0.3453**	0.0028
109	% of adults who reported having fair or poor health	**−0.4115**	0.0003
110	Average number of physically unhealthy days per month	**−0.3528**	0.0022
111	Average number of mentally unhealthy days per month	**−0.2351**	0.0452
113	Age-adjusted lung & bronchus cancer incidence rate per 100,000 people	**−0.2495**	0.0332

^†^ Serial number of assigned to various independent variables. ^§^ Bold style used to indicate significant correlation coefficients (*r*).

**Table 3:** A brief correlation matrix for a selected set of independent variables showing potential multicollinearity (all 1485 correlation coefficients among 55 independent variables are not shown for the sake of brevity, but a summary is presented in Table 4).

	Population Diversity Index	Residential Segregation Index ^†^	Median Age of the Population	% of Population Below 18 Years of Age	% of Population 65 Years and Older	Urban Population Density (per sq. Miles)	% of Occupied Housing Units	% of Owner-Occupied Housing Units	Median Home Value ($)	% of Housing Units Built 1970 or Earlier
Population diversity index	1									
Residential segregation index ^†^	−0.131	1								
Median age of the population	−0.482 **	−0.041	1							
% of population below 18 years of age	0.407 **	0.084	−0.659 **	1						
% of population 65 years and older	−0.470 **	−0.057	0.932 **	−0.829 **	1					
Urban population density (per sq. miles)	0.540 **	−0.099	−0.628 **	0.392 **	−0.593 **	1				
% of occupied housing units	0.365 **	−0.041	−0.729 **	0.729**	−0.810 **	0.450 **	1			
% of owner-occupied housing units	−0.265 *	−0.014	0.312 **	0.052	0.162	−0.312 **	−0.154	1		
Median home value ($)	0.005	−0.326 **	−0.075	0.069	−0.131	0.193	0.240 *	0.378 **	1	
% of housing units built 1970 or earlier	0.099	0.244 *	0.037	−0.056	0.098	0.112	−0.169	−0.506 **	−0.660 **	1

* 0.01 < *p* ≤ 0.05, 71 (73-2) df; ** *p* ≤ 0.01, 71 (73-2) df. ^†^ Residential Segregation Index between white and Black populations.

**Table 4:** A summary from the correlation matrix among the 55 independent variables that have significant bivariate correlations with the dependent variable (number of radon tests conducted per 1000 occupied housing units).

Correlation Coefficient, r (Absolute Value)	Number of Bivariate Combinations	Significance of Correlation Coefficient, r
>0.281	1015	*p* ≤ 0.01, 71 (73-2) df
0.230–0.281	76	0.01 < *p* ≤ 0.05, 71 (73-2) df
<0.230	394	*p* > 0.05, 71 (73-2) df
**Total ^§^**	**1485**	

**^§^ **Total number of bivariate combinations among the 55 socio-economic and public health independent variables.

**Table 5:** Multivariate regression analysis results of radon testing rates (independent) versus 55 socioeconomic and public health variables according to the least squares method.

Parameter Description	Coefficient	Standard Error	t-Value	*p* > |t|	VIF ^†^
**(Constant)**	32.20	2.80	11.523	0.000	
Population diversity index	25.79	41.09	0.628	0.553	216.2
Residential segregation index between Black & white populations	−2.94	4.18	−0.704	0.508	Infinity
Median age of the population	−79.38	43.44	−1.827	0.117	241.61
% of population below 18 years of age	8.70	27.00	0.322	0.758	93.35
% of population 65 years and older	52.78	43.19	1.222	0.267	238.85
Urban population density (per sq. miles)	5.14	20.73	0.248	0.812	55.02
% of occupied housing units	−12.81	21.16	−0.605	0.567	57.34
% of owner-occupied housing units	−18.29	44.96	−0.407	0.698	258.88
Median home value ($)	56.27	27.93	2.015	0.091	99.91
% of housing units built 1970 or earlier (older homes)	−46.92	37.84	−1.240	0.261	183.37
% of housing Units built 1990 or later (newer homes)	−39.59	28.68	−1.381	0.217	105.3
Average household size of owner-occupied unit	−20.43	16.58	−1.232	0.264	35.22
Average household size of renter-occupied unit	0.25	16.70	0.015	0.989	35.73
% of single-family homes detached	0.09	168.74	0.001	1	3645.96
% of single-family homes, total: detached + attached	87.16	149.00	0.585	0.58	2842.87
% of units in mobile homes	56.94	51.11	1.114	0.308	334.49
Lit_P1 (proportion of population with literacy level P1 and below)	−559.58	1724.10	−0.325	0.757	380,627.4
Lit_P2 (proportion of population with literacy level at P2)	−523.25	383.96	−1.363	0.222	Infinity
Lit_P3 (proportion of population with literacy level P3 and above)	578.58	1193.92	0.485	0.645	Infinity
Proportion of population with literacy level P2 + P3 combined	528.18	1946.45	0.271	0.795	Infinity
Lit_A (literacy average score indirect estimates)	−125.48	511.39	−0.245	0.814	33,487
Num_P1 (proportion of population with numeracy level P1 and below)	517.85	1738.56	0.298	0.776	387,039.2
Num_P3 (proportion of population with numeracy level P3 and above)	−934.98	573.56	−1.630	0.154	42,124.48
Proportion of population with numeracy level P2 + P3 Combined	−421.19	2054.09	−0.205	0.844	540,274.3
Num_A (numeracy average score indirect estimates)	221.73	824.40	0.269	0.797	87,026.01
Less_HS (proportion of population with education less than high school)	727.57	1258.44	0.578	0.584	202,785.8
HS (proportion of population with high school education)	575.85	1262.06	0.456	0.664	203,956.1
More_HS (proportion of population with education beyond high school)	720.83	1896.37	0.380	0.717	460,487.4
% of those with some college education	16.63	39.38	0.422	0.687	198.59
% of those with a bachelor’s degree in population 25 years and older	5.10	33.38	0.153	0.884	142.68
Unemployment (proportion of population aged 16–64 who are unemployed)	4.60	8.63	0.533	0.613	9.54
Occupation: management (proportion of population age 16 and over who are in the labor force in management, business, science, and arts occupations)	−38.27	32.17	−1.190	0.279	132.5
Median household income	−19.64	36.12	−0.544	0.606	167.08
Childcare cost burden (% of household income spent/required for childcare expenses)	−2.01	23.31	−0.086	0.934	69.56
% of children in poverty	8.18	27.99	0.292	0.78	100.28
Poverty_100 (proportion of population who are below 100 percent of the poverty level)	−57.86	81.43	−0.711	0.504	849.12
Poverty_150 (proportion of population who are below 150 percent of the poverty level)	−25.49	33.41	−0.763	0.474	142.93
SNAP (proportion of households that have received Food Stamps/SNAP in the past 12 months)	−39.42	37.76	−1.044	0.337	182.61
Resilience: % of population with 1–2 risk factor	−12.16	14.33	−0.849	0.429	26.3
% of children in single-parent households	−4.04	28.74	−0.141	0.893	105.76
Social association rate (# of social associations per 10,000 population)	7.48	11.84	0.632	0.551	17.94
% of households with a computer	−48.30	34.87	−1.385	0.215	155.69
% of households with broadband internet subscription/access	20.13	26.44	0.761	0.475	89.48
Primary Care Physician (PCP) rate (#PCP per 100,000 population)	14.17	21.78	0.650	0.539	60.75
Primary Care Physician (PCP) ratio (#people served by one PCP)	−4.60	18.39	−0.250	0.811	43.32
Life expectancy	−38.43	26.95	−1.426	0.204	93
Food environment index (indicator of access to healthy foods: 0 is the worst, 10 is the best)	−25.28	19.40	−1.303	0.24	48.21
% of adults with obesity	−10.52	16.43	−0.640	0.546	34.57
% of population physically inactive	−44.76	40.75	−1.098	0.314	212.62
% of population with excessive drinking	1.40	19.20	0.073	0.944	47.22
% of adults who reported currently smoking	−34.77	55.34	−0.628	0.553	392.11
% of adults who reported having fair or poor health	53.49	70.09	0.763	0.474	629.01
Average number of physically unhealthy days per month	−50.99	52.81	−0.966	0.372	357.14
Average number of mentally unhealthy days per month	22.04	20.16	1.093	0.316	52.02
Age-adjusted lung & bronchus cancer incidence rate per 100,000 people	20.90	14.98	1.395	0.212	28.73

^†^ VIF: Variance Inflation Factor. **Notes:** R-squared: 0.945; Adjusted R-squared: 0.444; F-statistic: 1.885; Probability (F-statistic): 0.217; Omnibus statistic: 1.657; Probability (Omnibus): 0.437; Durbin–Watson: 2.111; The smallest eigenvalue: 1.26 × 10^−29^; Kaiser–Meyer–Olkin (KMO) statistic: 0.86.

**Table 6:** Component loadings from factor analysis ^¶^.

Variable Description	Variable Label	PAF ^†^-1	PAF-2	PAF-3	PAF-4	Communality
Median home value ($)	**Housing Characteristics**	**0.923**	0.049	−0.141	0.192	0.894
% of housing units built 1970 or earlier (older homes)		**−0.663**	0.039	−0.198	−0.416	0.643
% of housing units built 1990 or later (newer homes)		**0.646**	−0.032	0.255	0.404	0.634
% of single-family homes, total: detached + attached)		**0.650**	0.147	0.535	0.386	0.782
% of units in mobile homes		**−0.735**	−0.520	−0.135	−0.032	0.689
Lit_P1 (literacy level P1 and below)	**Population Literacy and Numeracy**	**−0.939**	0.138	−0.034	−0.005	0.937
Lit_P2 (literacy level at P2)		**−0.880**	−0.398	0.114	0.008	0.884
Lit_P3 (literacy level P3 and above)		**0.971**	0.089	−0.035	−0.001	0.984
Literacy level P2 + P3 combined		**0.939**	−0.139	0.032	0.005	0.937
Lit_A (literacy average score indirect estimates)		**0.990**	0.001	0.020	−0.028	0.982
Num_P1 (numeracy level P1 and below)		**−0.951**	0.145	−0.100	0.038	0.983
Num_P3 (numeracy level P3 and above)		**0.990**	0.091	−0.032	−0.021	0.971
Numeracy level P2 + P3 combined		**0.952**	−0.150	0.099	−0.027	0.985
Num_A (numeracy average score indirect estimates)		**0.976**	−0.060	0.077	−0.053	0.986
Less_HS (education less than high school)	**Institutional Education Attainment**	**−0.850**	−0.133	0.140	−0.171	0.764
HS (high school education)		**−0.859**	−0.410	0.170	0.003	0.887
More_HS (education beyond high school)		**0.946**	0.313	−0.183	0.091	0.988
% of those with some college education		**0.878**	0.321	−0.034	0.034	0.808
% of those with a bachelor’s degree in population 25 years and older		**0.856**	0.414	−0.197	0.142	0.914
Occupation: management (proportion of population aged 16 and over who are in the labor force in management, business, science, and arts occupations)	**Type of Employment**	**0.858**	0.305	−0.174	0.069	0.826
Median household income	**Employment, Economy, and Poverty**	**0.893**	0.286	0.239	0.259	0.878
Childcare cost burden (% of household income spent/required for child care expenses)		**−0.473**	0.462	−0.150	−0.040	0.556
% of children in poverty		**−0.891**	−0.099	−0.408	−0.047	0.920
Poverty_100 (proportion of population who are below 100 percent of the poverty level)		**−0.823**	0.025	−0.328	−0.209	0.824
Poverty_150 (proportion of population who are below 150 percent of the poverty level)		**−0.887**	−0.017	−0.247	−0.210	0.870
SNAP (proportion of households that have received Food Stamps/SNAP in the past 12 months)		**−0.874**	0.008	−0.008	−0.228	0.804
% of households with a computer	**Access to Computer and Internet**	**0.840**	0.349	0.275	−0.099	0.787
% of households with broadband internet subscription/access		**0.849**	0.282	0.236	−0.110	0.772
Primary Care Physician (PCP) rate (#PCPs per 100,000 population)	**Access to Healthcare, Health & Well-being, and Lifestyle**	**0.484**	0.244	−0.333	−0.034	0.425
Life expectancy		**0.857**	0.186	−0.142	0.311	0.845
Food environment index (indicator of access to healthy foods: 0 is the worst, 10 is the best)		**0.717**	−0.143	0.109	0.101	0.586
% of adults with obesity		**−0.798**	0.185	0.054	0.146	0.736
%of population physically inactive		**−0.975**	0.012	0.021	−0.014	0.954
% of population with excessive drinking		**0.548**	−0.510	−0.009	−0.337	0.752
% of adults who reported currently smoking		**−0.944**	−0.336	0.060	−0.180	0.959
% of adults who reported having fair or poor health		**−0.981**	0.035	−0.063	−0.007	0.976
Average number of physically unhealthy days per month		**−0.968**	−0.189	−0.016	−0.161	0.940
Average number of mentally unhealthy days per month		**−0.770**	−0.412	0.027	−0.304	0.764
Age-adjusted lung & bronchus cancer incidence rate cases per 100,000 people		**−0.571**	−0.340	0.343	−0.456	0.774
% of children in single-parent households	**Social Factors**	**−0.680**	0.159	−0.355	0.185	0.715
Population diversity index	**Demographic & Neighborhood Characteristics**	−0.003	**0.853**	0.039	0.253	0.770
Median age of the population		−0.247	**−0.855**	−0.478	0.351	0.865
% of population 65 years and older		−0.317	**−0.857**	−0.666	0.274	0.964
Urban population density (per sq. miles)		0.277	**0.742**	0.009	−0.230	0.631
% of occupied housing units	**Housing Characteristics**	0.456	**0.649**	0.572	−0.251	0.739
% of population below 18 years of age	**Demographic & Neighborhood Characteristics**	0.264	0.685	**0.783**	−0.067	0.797
Average household size of owner-occupied unit	**Housing Characteristics**	0.376	0.551	**0.744**	0.158	0.718
Average household size of renter-occupied unit		0.101	0.323	**0.680**	0.049	0.440
% of single-family homes detached		0.562	0.051	**0.568**	0.395	0.748
% of owner-occupied housing units	**Housing Characteristics**	0.343	−0.360	0.289	**0.520**	0.680
**Variance**		**28.947**	**5.551**	**3.971**	**2.205**	**40.674**
**% of variance**		**57.89**	**11.10**	**7.94**	**4.41**	**81.35**
**Cronbach’s alpha (standardized)**		**0.988**	**0.882**	**0.818**	**N/A ^‡^**	

^¶^ Bold style used to indicate the highest loading in any given row. ^†^ Principal axis factor. **^‡^** Not Applicable.

**Table 7:** Regression analysis results based on the factor analysis results.

	Coefficients	Standard Error	t-Value	*p* > |t|	VIF ^§^	Cronbach’s Alpha (Standardized)
Constant (b_0_)	37.380	3.427	10.907	0.000	1.000	
PAF ^†^-1	12.570	4.562	2.756	0.008	1.770	0.988
PAF-2	−37.726	7.480	−5.043	0.000	4.760	0.882
PAF-3	−20.454	9.494	−2.154	0.035	7.680	0.818
PAF-4	6.985	6.344	1.101	0.275	3.430	N/A ^¶^

^†^ Principal axis factor. **^§^** VIF: Variance Inflation Factor. **^¶^ Not Applicable**; **Notes:** R-squared: 0.362; Adjusted R-squared: 0.321; F-statistic: 8.925; Probability (F-statistic): 8.94 × 10^−6^.

## Data Availability

The data presented in this study are available on request from the corresponding author, contingent on privacy restrictions. Approval will be subject to meeting the University’s Ethics processes, as well as signing a Non-Disclosure Agreement.
